# Selenium in cancer management: exploring the therapeutic potential

**DOI:** 10.3389/fonc.2024.1490740

**Published:** 2025-01-07

**Authors:** Lingwen He, Lu Zhang, Yulong Peng, Zhijun He

**Affiliations:** ^1^ Department of Oncology, Dongguan Songshan Lake Tungwah Hospital, Dongguan, China; ^2^ Department of Oncology, Dongguan Tungwah Hospital, Dongguan, China; ^3^ School of Modern Industry for Selenium Science and Engineering, Wuhan Polytechnic University, Wuhan, China; ^4^ Shenzhen Key Laboratory of Marine Biotechnology and Ecology, College of Life Sciences and Oceanography, Shenzhen University, Shenzhen, Guangdong, China

**Keywords:** selenium, cancer, selenoprotein, selenium compounds, selenium nanoparticles

## Abstract

Selenium (Se) is important and plays significant roles in many biological processes or physiological activities. Prolonged selenium deficiency has been conclusively linked to an elevated risk of various diseases, including but not limited to cancer, cardiovascular disease, inflammatory bowel disease, Keshan disease, and acquired immunodeficiency syndrome. The intricate relationship between selenium status and health outcomes is believed to be characterized by a non-linear U-shaped dose-response curve. This review delves into the significance of maintaining optimal selenium levels and the detrimental effects that can arise from selenium deficiency. Of particular interest is the important role that selenium plays in both prevention and treatment of cancer. Finally, this review also explores the diverse classes of selenium entities, encompassing selenoproteins, selenium compounds and selenium nanoparticles, while examining the mechanisms and molecular targets of their anticancer efficacy.

## 1 Introduction

Cancer condition is a formidable public health issue that afflicts millions of individuals across the globe, with an increasing number of cases being reported annually. The most prevalent forms of this disease within the population are lung, breast, colorectal and prostate cancers (1, 2). The heterogeneity of cancers is increasingly being acknowledged as a pivotal characteristic in the field of oncology (3). The incidence and mortality rates of these cancers vary significantly by time, geographic region, age, and gender. For instance, according to the Global Cancer Statistics 2020, lung cancer remains the most common cancer worldwide, with the highest incidence rates in certain regions such as East Asia and Europe (4). Breast cancer is the most frequently diagnosed cancer among women, with nearly 2.3 million new cases reported in 2020, and it disproportionately affects developed countries (4, 5). Colorectal cancer is also a leading cause of cancer-related mortality, with the highest rates observed in North America and Europe (6). Prostate cancer is the second most common cancer among men globally, with significant variations in incidence rates across different populations, being particularly high in developed countries (7). Understanding these disparities is crucial for targeted prevention and treatment strategies.

Alterations in the equilibrium of micronutrients can potentially contribute to the process of tumor formation by exacerbating cellular damage, inducing DNA lesions, and disrupting the delicate balance of cellular redox status (8). In addition, trace minerals are recognized for their salutary effects on a range of biological pathways. They play a crucial role in cell stabilization, the modulation of oxidative stress, the intricate DNA damage response (DDR)/repair mechanisms, as well as serving as potent antioxidants (9). For instance, zinc, an integral component of the antioxidant enzyme superoxide dismutase (SOD), effectively neutralizes free radicals and subsequently stimulates the activity of DNA repair enzymes (10). This cascade of events serves as a formidable anticancer defense mechanism. In a parallel vein, selenium, a trace element of paramount importance, is indispensable for the optimal maintenance of human health and well-being (11). Selenium, a cornerstone of selenoenzymes, plays a pivotal role in mitigating DNA damage, curbing oxidative stress, alleviating inflammation, and facilitating the detoxification of carcinogenic substances (12). This vital trace element is indispensable for the formation of selenocysteine, a genetically encoded amino acid, and is crucial for the genesis of a suite of proteins called selenoproteins. Selenium’s protective powess against the onslaught free radicals is attributed to its strategic positioning within active center of antioxidant enzymes, including the renowned glutathione peroxidase, thereby fortifying the body’s defense against oxidative harm (11, 13–15). Selenium exerts a profound influence on the immunological system, bolstering the immune response across both the innate and acquired immune systems (16). This trace element is instrumental in amplifying the efficacy of the immune system, particularly in the realms of cytotoxic lymphocyte function and the dynamic activity of natural killer cells (17). In our daily diet, selenium can be predominantly sourced from a variety of nutritious foods, including whole grains, an array of vegetables, a selection of seafood, various types of meat, dairy products and nuts (18). This essential micronutrient is delivered to the body via both natural food sources and dietary supplements, and it exists in two principal forms: the organic forms, which include selenomethionine and selenocysteine, and the inorganic forms, which encompass selenite and selenate. Upon absorption into the body, both organic and inorganic forms undergo a transformation into selenides, which are integral to the synthesis of selenoproteins (19). Selenium boasts a diverse array of functions that contribute to overall health. It serves as a powerful antioxidant, combats inflammation, bolsters the immune system, and plays a significant role in diminishing the risk of cancer. Additionally, selenium exhibits properties that inhibit the invasive and metastatic capabilities of tumor. Beyond these preventative measures, selenium also finds application in the clinical realm, offering support in the treatment of cancer through radiation therapy and chemotherapy protocols. Numerous scientific publications have consistently demonstrated a significant correlation between selenium levels in human serum and the incidence of various types of cancer. This association has been meticulously investigated across a spectrum of malignancies, including but not limited to cancers of the colon, lung, bladder, breast, ovary and prostate. Consequently, the recognition of selenium’s potential in cancer prevention and treatment has led to a surge in its global popularity (20–24).

Nevertheless, it is imperative to approach selenium with a discerning eye, as it embodies a dual nature that can be both beneficial and detrimental. The balance is delicate; both deficiencies and excessive intakes of selenium can induce harmful effects on the body. Selenosis, a condition arising from an adequate or marginally excessive selenium intake, can pose significant risks to individuals (25). Selenium, a trace element of paramount importance, walks the fine between being an indispensable nutrient and a potential toxin. Given these complexities, the chemopreventive role of selenium in cancer remains a subject of ambiguity. Consequently, there is a pressing need for more comprehensive and meticulous research to unravel the intricate relationship between selenium and its impact on health. Further studies are essential to clarify the nuances of selenium’s role and to establish guidelines for its safe and effective use in promoting wellness and preventing disease. It is equally important to recognize that the effectiveness and outcomes of such research are likely contingent upon several variables. These include the specific chemical form of the Se compound in question, whether it be inorganic, such as sodium selenite, or organic, such as selenomethionine (SeMet). Additionally, the dosage administered, the compound’s bioavailability, the baseline selenium levels within the study cohort, the particular type of cancer under investigation, and even the stage of the cancerous lesion can all significantly influence the results (26, 27). A comprehensive understanding of these factors is essential for interpreting the study’s findings and for guiding future research in this area.

This comprehensive review delves into the role of selenium in both the prophylaxis and therapeutics of cancer. It encapsulates an overview of the current understanding regarding selenium’s contribution to oncological management, encompassing its therapeutic modalities. Additionally, it presents a synthesis of the principal findings from the extant literature that investigates the nexus between selenium and cancer, placing particular emphasis on the most contemporary outcomes that have marked significant advancements in the research domain. Ultimately, the review meticulously compiles pertinent Se species and scrutinizes their emergent progress and prospective efficacy in the realm of cancer prevention and treatment. This scholarly work paves the way for forthcoming investigative endeavors in this critical field.

## 2 Metabolism and biological effects of selenium

**Figure 1:**
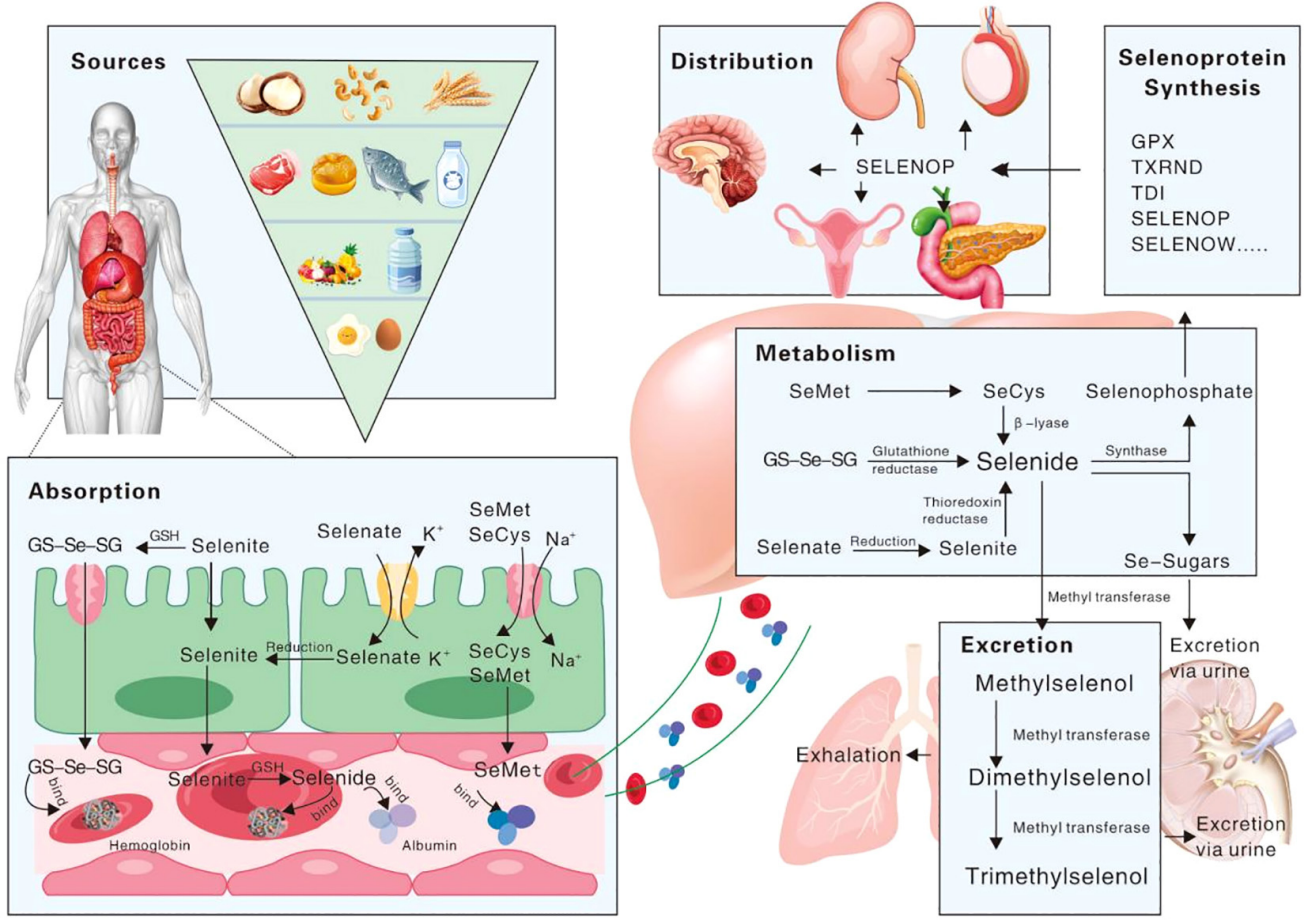
Schematic representation of selenium’s biological process in the human body.

Selenium metabolism is a complex and systemic biological process involving selenium absorption, transport, transformation, and excretion, as illustrated in **Figure 1**. Selenium is obsorbed from the diet in both organic forms, such as selenomethionine (SeMet) and selenocysteine (Sec), and inorganic forms, including selenite and selenate. The absorption of selenium and its associated compounds predominantly takes place in the duodenum and small intestine, a process that is mediated by an array of transport mechanisms. It is noteworthy that the efficiency of absorption varies among the diverse selenium species, reflecting the nuanced complexity of this metabolic pathway. Although elemental selenium, selenium dioxide, and selenium sulfide exhibit limited bioavailability, compounds such as selenite, selenate, and selenium-enriched amino acid analogs are more amenable to absorption, particularly when synergized by vitamins A, D and E (28, 29). The direct uptake of selenite typically does not surpass 60%; the majority of this is converted into seleno-substituted diglutathione (GS-Se-SG) in the intestinal lumen, thereby enhancing its absorption potential (30). A minor fraction of selenite and GS-Se-SG undergoes metabolic conversion to selenide, which preferentially binds with albumin and hemoglobin, subsequently being conveyed to the liver (31). Post-absorption, SeMet is also directed to the liver, facilitated by albumin. Nevertheless, the intricate details of the transport mechanisms for selenate and selenocysteine (Sec) remain somewhat enigmatic, warranting further investigation.

Selenium is metabolized through the liver, and synthesized and exported to selenoprotein P (SelP), which is eventually secreted into the bloodstream, serving as a vital conduit for the distribution of selenium to peripheral tissues or organs (32, 33). SelP stands out among selenoproteins due to its distinctive C-terminal structural domain, which is replete with multiple Sec residues that facilitate extracellular selenium transport (34, 35). Once transported, selenium is then converted to selenophosphate through an intricate intracellular selenometabolic pathway. The excretion of selenium from the human body is a meticulous process, primarily occurring through the exhalation of breath or the elimination of urine. During this process, selenium is converted into small molecule metabolites, which are formed through a series of methylation reactions (36–38).

The biological effects of Se are exerted primarily through the form of selenoproteins synthesized by the selenometabolic system. Once inorganic selenium enters the cell, it may be reduced to hydrogen selenide (H_2_Se) by glutathione (GSH) (39) or thioredoxin (TXN) (40). Selenide can then be transformed into selenocysteine (Sec) through the catalytic action of cysteine synthase, or alternatively, through a coordinated enzymatic process involving selenohydrosine dikinase, Se-cysteine-tRNA synthase, and cysteine-tRNA ligase. This intricate biochemical pathway ensures the precise incorporation of Sec into the protein structure. The subsequent step involves the delivery of selenocystyl-tRNA, which carryies Sec, to the UGA which is normally a stop codon. However, in the synthesis of selenoproteins, the UGA codon is uniquely recognized as a codon for Sec. In response to this codon, Sec is co-translationally integrated into the nascent polypeptide chain (41). This exceptional mechanism exemplifies the sophisticated regulation of the genetic code, allowing for the seamless incorporation of Sec into selenoproteins, which are crucial for a myriad of biological functions. Proteins containing selenocysteine are collectively known as selenoproteins.

Selenoproteins are a unique class of proteins containing the rare amino acid selenocysteine (Sec), often hailed as the 21st proteinogenic amino acid. The biological functions of selenium are mainly exerted through the structural domains of selenoproteins containing Sec residues. As of the latest research, the human genome has revealed the presence of 25 distinct selenoprotein genes (42). Furthermore, selenoproteins can be categorized into several subfamilies according to their cellular functions, including subfamilies involved in antioxidant defense, redox regulation, thyroid hormone metabolism, selenium transport and storage, selenophosphate synthesis, calcium metabolism, myogenesis, protein folding and protein amination (43, 44).

## 3 Selenium and cancer prevention

Alterations in redox homeostasis are thought to be a contributing factor in the pathogenesis of numerous diseases, especially cancer. This is because oxidative damage can result in genomic instability, DNA mutations, and tumorigenesis and progression. Selenium, known for its antioxidant properties through the action of selenoproteins, has been postulated to mitigate oxidative stress. Consequently, it has been theorized that selenium supplementation could potentially thwart the onset and progression of cancer. Supporting this hypothesis, animal tumor models have shown that selenium supplementation can decrease the occurence and severity of liver, esophageal, pancreatic, prostate, colon, and breast cancers (45–49).

Concentrated efforts to prevent cancer by selenium has its roots in the late 1960s. In a pioneering study conducted in 1966, Shamberger and Rudolph demonstrated that sodium selenide (Na_2_Se) drastically curtailed the development and expansion of tumors in a chemically induced mouse skin model (50). In the United States, the notion that selenium might possess anticancer properties was further bolstered by empirical observations suggesting an inverse correlation between cancer mortality rates and selenium concentrations in both blood and feed crops (51, 52). Since the 1970s, a plethora of epidemiological studies and clinical trials focusing on selenium supplementation have lent credence to the “selenium cancer hypothesis.” This hypothesis posits that a diet low in selenium associated with an elevated risk of developing cancer. Collectively, these investigations have reinforced the idea that selenium’s role in cancer prevention is not merely speculative but is supported by a substantial body of evidence.

**Figure 2:**
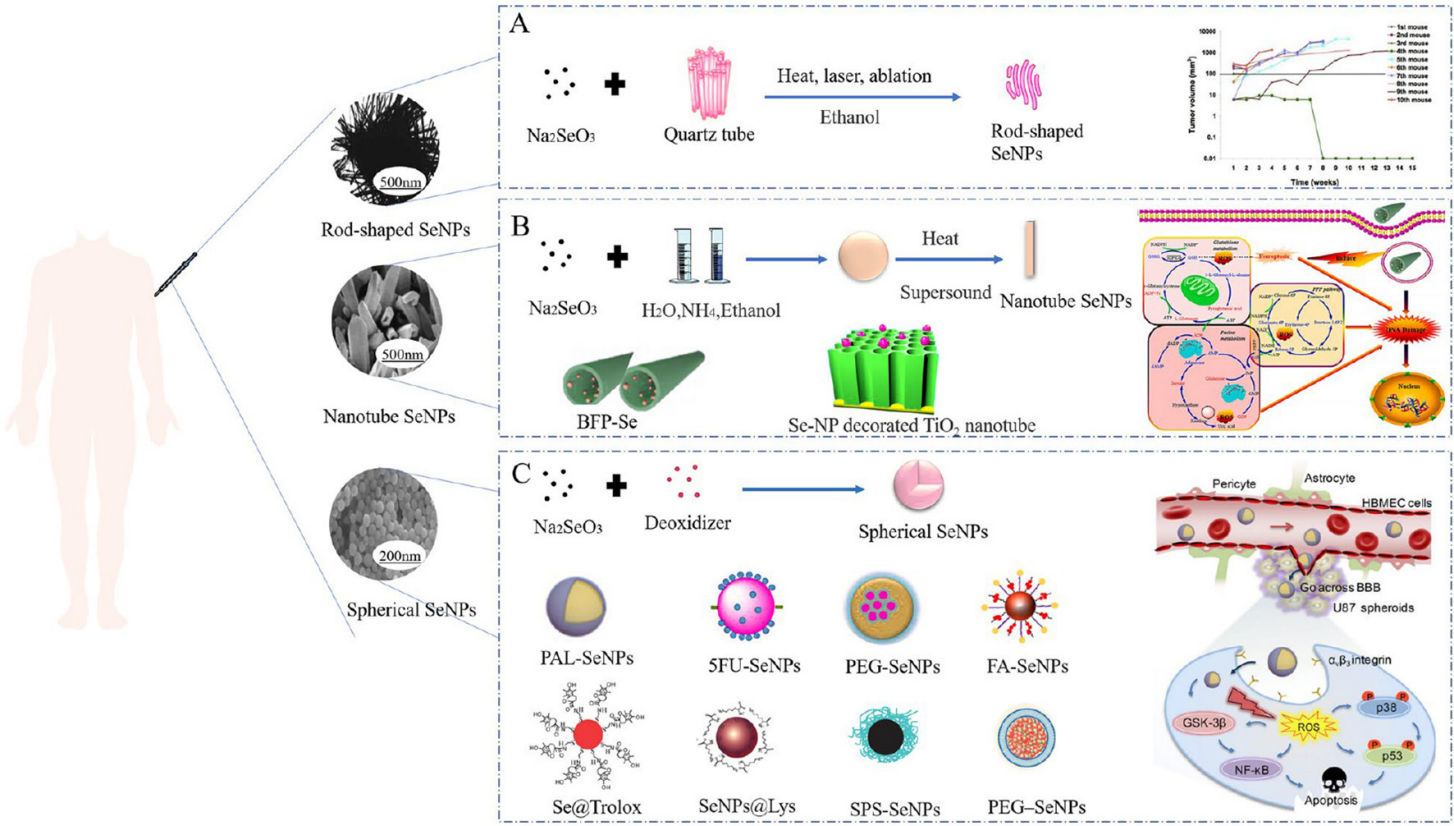
Morphology of diferent SeNPs used in cancer treatment and their biological application (53). **(A)** Sodium selenite and quartz tubes were treated with alcohol by heat, laser, and ablation to synthesize rod-shaped SeNPs. **(B)** Nanotube SeNPs were synthesized with sodium selenite, water, ammonia and ethanol by heating and sonication. **(C)** Synthesis of spherical SeNPs from sodium selenite and potato extract.

In comparison to inorganic and organic compounds (in which inorganic forms are more harmful than organic ones), SeNPs have gained considerable attention due to their high bioavailability, strong biological activity, and low toxicity. More suitable items have been created using nanotechnology to guarantee their physiological and therapeutic efects. SeNPs have a wide variety of biological applications, have been developed for dietary supplements as well as therapeutic agents and do not exhibit noticeable side efects in cancer. According to the fndings of many researchers, SeNPs are benefcial in the chemoprevention of cancer as a potential anticancer medication and carrier of anticancer drugs. Moreover, the size and morphology of SeNPs can afect their biological activity and uptake capacity of cells (53). Terefore, it is very important to select the appropriate method for preparing the desired nano size and morphology of SeNPs (**Figure 2**).

### 3.1 Selenium and breast cancer

Breast cancer, a malignant neoplasm that originates in the glandular epithelium of the mammary gland, holds the distinction of being the most prevalent form of cancer affecting women across the globe. The age-standardized incidence rate of prostate cancer was 11.6% in developed countries, which ranked second following lung cancer. The global cancer statistics for the year 2020 show that approximately 2.3 million new cases of breast cancer, accounting for 11.7% of all female cancer diagnoses, are identified worldwide (4). Breast cancer is anticipated to remain one of the primary contributors of female mortality in Europe (54) and is projected to rank as the fifth leading cause of cancer-related deaths on a global scale (55). So far, research into the protective role of selenium against breast cancer has been limited, yielding inconsistent findings that warrant further investigation.

In their study, Martin et al. observed that selenate effectively enhanced trans-epithelial electron resistance and substantially decreased paracellular permeability to macromolecules in breast cancer cells (56). And under selenate’s influence, the metastatic MDA-MB-231 breast cancer cells exhibited markedly diminished motility and a reduced capacity to breach the endothelial cell barrier. Furthermore, in addition to impacting endothelial cells and curtailing the production of matrix metalloproteinase-2 (MMP-2), methylated selenium has also been shown to inhibit vascular endothelial growth factor (VEGF) expression in breast cancer cells (57). Suzana and coworkers have documented a correlation indicating that the risk of breast cancer diminishes progressively with each ascending quartile of selenium intake (58). In a separate study, Harris and colleagues reported a notably inverse relationship between dietary selenium consumption and the risk of breast cancer-specific mortality in Sweden, a nation characterized by relatively low selenium intake levels. And they also found that an elevated selenium intake prior to breast cancer diagnosis has contributed to improve specific survival rate, as evidenced by a hazard ratio of 0.69 (95% confidence interval: 0.52-0.92), and clinical outcomes (59). In 2021, Szwiec et al. conducted a cohort study of 538 breast cancer patients in Poland and showed that patients with lower serum selenium levels at the time of diagnosis faced a heightened risk of mortality over the subsequent decade (60). In contrast, a comprehensive prospective case-cohort study involving 145,033 postmenopausal women in the United States, which included 9,487 cases of breast cancer, did not uncover any correlation between daily dietary selenium intake and the risk of developing breast cancer (61). Bengtsson and colleagues conducted a comparative analysis of varying levels of selenium intake in relation to breast cancer incidence, but no significant difference in breast cancer risk was observed between the groups examined (62). Similarly, a study from Spain reported no discernible protective effect of dietary selenium intake on the incidence of breast cancer (63). The data regarding the relationship between selenium levels and the risk of breast cancer, as well as patient survival, are indeed inconsistent. The disparity in these findings can be attributed to the baseline selenium levels present within the populations being studied (64, 65).

The widespread feature of breast cancer is heterogeneity. Since the 19th century, breast tumor heterogeneity has been observed and described, and these differences have become the basis of disease classification (66). The spatial and temporal differences in heterogeneity, phenotype and genotype between and within tumors have a significant impact on the clinical management of breast cancer patients, affecting prognosis and treatment response (67). As an antioxidant, the role of selenium in the treatment of breast cancer may be affected by tumor heterogeneity. For example, selenium may enhance the effect of chemotherapy in some subtypes, but the effect is not obvious or resistant in other subtypes. This requires that in the clinical application of selenium as an adjuvant therapy, the specific biological characteristics and subtypes of the tumor need to be considered to achieve individualized treatment.

### 3.2 Selenium and lung cancer

Lung cancer has emerged as one of the most prevalent forms of malignant tumors and ranks highest in terms of both incidence and mortality worldwide. The conventional therapeutic arsenal comprises surgery, chemotherapy and radiation therapy. Despite rapid progress over the past decade, there are still inherent limitations to these treatments. Therefore, there is an urgent need to identify treatments that are both safer and more efficacious, prompting ongoing research aimed at uncovering drugs that are more potent and less toxic. In recent times, the identification of antitumor agents derived from the essential trace element selenium has presented promising avenues for the management of lung cancer.

Lung cancer’s heterogeneity, spanning cellular and histological levels, profoundly influences pathogenesis, diagnosis, molecular diagnostic tissue selection, and treatment decision-making (68). This heterogeneity is not only a hallmark of the disease but also a critical factor in determining the efficacy of treatments, including selenium supplementation. The impact of selenium on lung cancer treatment and outcomes is multifaceted and contingent upon the tumor’s unique characteristics. In a subsequent investigation, Reid delved into the relationship between selenium supplementation and lung cancer incidence, revealing that the intake of selenium supplements was associated with a reduced risk of lung cancer, exhibiting a negative correlation with its incidence (69). It has been documented that selenium-enriched yeast can reduce metastasis in a murine model of Lewis lung carcinoma (70). The research also indicated patients suffering from malignant pleural effusion as a result of advanced lung cancer tend to have diminished serum selenium levels in comparison to those of healthy individuals (71). This finding further underscores the significance of Se in the context of lung cancer. The selenoprotein glutathione peroxidase 4 (GPx4) is an important negative regulator of iron death (72). The application of GPx4 siRNA or the GPx4 inhibitor Rsl-3 has been shown to markedly suppress the proliferation and migration of lung cancer cells. Notably, this inhibitory effect can be counteracted by Fer-1, an inhibitor of ferroptosis, a form of iron-dependent cell death. This observation implies that the selenoprotein GPx4 holds promise as a novel therapeutic target for the treatment of lung cancer (73, 74). Selenium can slow down the side effects of lung cancer after radiation and chemotherapy. A study by Mix et al. included 16 patients with non-small cell lung cancer who were given organic selenium one week prior to concurrent radiation and chemotherapyand showed that selenium supplementation helped to reduce treatment-related side effects such as myelosuppression (75).

So far, not all patients with lung cancer can benefit from the treatment of each stage of the disease. The development of drug resistance, adverse events, and inevitable disease progression highlights the urgent need for new diagnosis, prognosis, and treatment options for these diseases. The heterogeneity of lung cancer has an impact on the understanding of pathogenesis, diagnosis, molecular diagnostic tissue selection and treatment decision-making. The supplement and application of selenium need to be individualized according to the specific type and stage of lung cancer and the individual differences of patients. At the same time, the dose, form of selenium and its combination with other treatment methods need further research to clarify its effect and optimal application strategy in the treatment of lung cancer.

### 3.3 Selenium and thyroid cancer

The thyroid gland is pivotal in maintaining homeostasis, fostering growth and development, and ensuring the proper functioning of the reproductive, nervous and cardiovascular systems. There has been a striking global increase in the incidence of thyroid cancer over the past 30 years, outpacing the rise of any other form of cancer (76, 77). Beyond unchangeable external factors such as age, gender, ethnicity, and genetic predisposition to thyroid cancer, the influence of chemical elements on thyroid malignancy has garnered significant research interest. The normal operation of the thyroid gland hinges on an array of trace elements that are indispensable for the synthesis and metabolism of thyroid hormones (78–80). Selenium is not only an integral component of the synthesis and metabolism of thyroid hormones but also plays a unique role in the thyroid gland (81). Thyroid hormone metabolism is mediated by 3 iodothyronine deiodinases which are selenoproteins (82–84). Selenium stands out as a crucial element for both the production and the activity of thyroid hormones (85). It is found that the the thyroid gland, which exhibits the highest selenium concentration per unit of tissue, remarkably retains selenium and continues to express selenoproteins even in the face of severe selenium deficiency, reflecting the distinctiveness of the human thyroid and highlights the critical importance of selenium for its proper functioning and health (86–88). Beyond their role in thyroid hormone metabolism, selenoproteins are indispensable for a multitude of vital cellular processes. They are essential in the detoxification of tissue-damaging peroxides, the reduction of oxidative stress on proteins and cell membranes, the modulation of intracellular redox signaling pathways, and the maintenance of thyroid hormone homeostasis. These functions underscore the multifaceted significance of selenoproteins in safeguarding cellular integrity and supporting the intricate balance of thyroid hormone activity (89).

Numerous clinical investigations have demonstrated a substantial correlation between serum selenium concentrations and the presence of thyroid cancer. These studies consistently reveal that individuals diagnosed with thyroid cancer tend to exhibit lower serum selenium levels in comparison to those observed in healthy control subjects (90–93). A study conducted in 2019 indicated that a deficiency in selenium elevates the risk of developing hyperthyroidism in patients with Graves’ disease and those suffering from nodular goiter. However, the same study found that selenium supplementation did not have a significant impact on the levels of TSH receptor autoantibodies or on the proliferation of T-cells (94). Appropriate selenium supplementation combined with thyrotazole therapy results in faster recovery from hyperthyroidism than thyrotazole therapy alone (95). Several observational studies propose that Se may exert an influence on the trajectory of autoimmune thyroid diseases by modulating the immune response (96, 97). A prospective study conducted by Xu, X., and his team revealed no substantial association between Se intake and thyroid cancer (98). This finding contrasts with prior research that has explored the link between reduced Se levels and thyroid cancer. In light of this discrepancy, we postulate that the incongruity of the study’s results may be attributable to methodological biases in the assessment of selenium levels. Although the incorporation of selenium-enriched compounds into the therapeutic regimen for thyroid cancer has not yet been endorsed, their potential anticancer effects have garnered considerable interest. It is important to emphasize that the application of selenium-containing compounds as an intervention in cancer development and progression warrants meticulous evaluation.

The intricate relationship between selenium and thyroid cancer is influenced by a multitude of factors, with the individual’s baseline selenium levels and genetic profile being paramount. The heterogeneity of thyroid cancer encompasses a spectrum of pathological types, each with distinct molecular signatures and clinical behaviors (99). This diversity implies that various forms of thyroid cancer may exhibit different sensitivities and responses to selenium and its compounds. For instance, differentiated thyroid cancers, which include papillary and follicular carcinomas, may interact with selenium in a manner distinct from that of more aggressive forms such as anaplastic thyroid cancer.

In light of these considerations, a personalized medicine approach is warranted for selenium supplementation in thyroid cancer. This approach should take into account the patient’s genetic background, the tumor’s molecular characteristics, and the potential for selenium to interact with existing treatments, such as chemotherapies and radiotherapies. Future research endeavors should focus on elucidating the complex interactions between selenium and the diverse thyroid cancer subtypes, aiming to uncover the optimal conditions for selenium’s therapeutic potential and to develop strategies that maximize its efficacy while minimizing potential adverse effects.

### 3.4 Selenium and prostate cancer

Prostate cancer ranks as the second most common male malignancy globally and holds the top position in the United States (100). The etiology and advancement of this disease involve a intricate interplay of genetic and environmental determinants (101, 102). Selenium, a trace mineral with a spectrum of biological functions, has emerged as a focal point in prostate cancer research, holding promise for its potential in both prevention and therapeutic strategies. An increasing body of evidence suggest that selenium may act as a prophylactic agent, with circulating selenium levels potentially linked to the risk of prostate cancer development (103). Interestingly, both suboptimal and elevated selenium levels have demonstrated significant effects, indicating a nuanced relationship between selenium status and prostate cancer outcomes. A meta-analysis conducted by Cui et al. integrating results of 17 studies that measured selenium status by serum and toenail selenium observed an inverse relationship between serum selenium levels and prostate cancer risk (104). The present investigation, which involved the collection of plasma samples from 116 Caucasian men with delayed-onset prostate cancer in South Australia, along with 132 well-matched controls, indicated that the mean plasma selenium concentration was notably lower among individuals with prostate cancer compared to the control group (105). This finding seems to corroborate the abnormal expression of Se levels in prostate cancer. Conversely, findings from a cohort study encompassing 784 cases of prostate cancer and a corresponding control group indicated that the median plasma selenium levels and SelP concentrations were similar between the two groups, with a notable elevation in high-grade prostate cancer cases (106). These suggest that while plasma selenium status may not be directly linked to the overall risk of prostate cancer, there is an association between elevated selenium levels and a reduced risk of high-grade prostate cancer. In addition, recent studies have uncovered that genetic factors and single nucleotide polymorphisms (SNPs) within selenoprotein genes can modulate the body’s response to selenium, potentially playing a role in the prevention of prostate cancer (103, 107).

Based on current research, selenium supplementation alone has not shown favorable results in prostate cancer treatment. A study endeavored to evaluate the potential of selenium supplementation to decelerate the progression of prostate cancer by administering daily doses of selenium to patients with prostate-specific antigen (PSA) indicative of prostate cancer (108). The findings revealed that selenium supplementation did not exert a significant impact on the PSA velocity in cases of localized prostate cance. Another study evaluated mortality in 4,459 patients supplemented with selenium after diagnosis and found that supplementation of 140 μg/day or more after diagnosis of non-metastatic prostate cancer increased prostate cancer mortality (109). This may be due to the need to consider an individual’s selenium levels, as well as other influencing factors, and to supplement with appropriate doses of selenium.

While standalone selenium supplementation has not yet proven to positively influence the progression of prostate cancer, it has exhibited potential in augmenting the effectiveness of chemotherapy and radiation therapy, as well as in alleviating the side effects that typically accompany these treatments. For instance, sodium selenite has been demonstrated to substantially enhance the radiosensitization of HI-LAPC-4 and PC-3 xenograft tumors (110). In the realm of chemotherapy, the concomitant use of doxorubicin and sodium selenite has shown a synergistic inhibition of prostate cancer cell proliferation, surpassing the effects of doxorubicin in isolation (111). While selenium holds promise in prostate cancer management, the heterogeneity of the disease and the complexity of selenium’s interactions with various patient characteristics underscore the need for personalized approaches and further research. Future studies should consider baseline selenium status, age, genetic factors, and other variables to elucidate the activity of selenium in cancer prevention and to identify the subpopulations most likely to benefit from selenium supplementation. Therefore, it is imperative that additional research and clinical trials are undertaken to ascertain the most efficacious dosage, duration, and timing of selenium supplementation regimens within cancer therapeutic strategies.

### 3.5 Selenium and liver cancer

Primary liver cancer (PLC), which ranks as the sixth most prevalent cancer globally, is also the third leading cause of cancer mortality (4, 112). The incidence of PLC is steadily increasing. Hepatocellular carcinoma (HCC) and intrahepatic cholangiocarcinoma (ICC) constitute the predominant forms of PLC, collectively accounting for approximately 95% of all cases (113, 114). It was estimated that there were 905,677 new cases of PLC, resulting in nearly 830,180 fatalities worldwide in 2020 (4). The heterogeneity of PLC is not only reflected in its varying pathological types but also in its diverse responses to selenium supplementation. Both animal models and clinical research have highlighted the significance of adequate selenium intake and consistent selenoprotein synthesis rates in safeguarding hepatocytes from damage and in mitigating the risk of PLC. Epidemiological data indicates a heightened risk of PLC in regions with marginally lower selenium levels, such as in Western Europe (115).

A burgeoning corpus of research indicates that the diminished expression of selenoproteins may be a contributing factor to the risk of PLC. This is attributed to the weakening of the body’s inherent antioxidant defenses and the detrimental influence on immune system dynamics (116). Liver cells, or hepatocytes, are especially vulnerable to oxidative stress, inflammatory responses, hypoxic conditions, and endoplasmic reticulum stress. Notably, hepatic selenoprotein expression was also affected by these factors, suggesting an interrelated modulation between cancer protective and risk factors (116). Selenium deficiency causes systemic redox imbalance, and blood inflammation with liver pathology (117). In a case-control study in the European Prospective Investigation into Cancer and Nutrition cohort, researchers analyzed the relationship between pre-diagnostic selenium status and the risk of PLC. The findings indicated that elevated levels of Se and selenoprotein P were significantly associated with a reduced risk of HCC (118). Studies have shown that reduced selenium levels lead to the accumulation of lipid peroxides, which increase the activity of AP-1, and then upregulate the expression of VEGF and interleukin-8, thereby accelerating the growth of hepatocellular carcinoma cells (119). Furthermore, viral infections have the potential to significantly disrupt selenium metabolism and the biosynthesis of selenoproteins (120). The carcinogenic implications of such interactions are likely to be most pronounced in individuals with selenium deficiency, which may explain part of the observed correlation between low selenium levels and the increased risk of PLC. Overall, the existing body of evidence further implicates PLC in the expanding array of health concerns associated with selenium deficiency and call for more evidence-based discussion of the importance of selenium as a health-related micronutrient.

## 4 Anticancer mechanism of selenium

**Figure 3:**
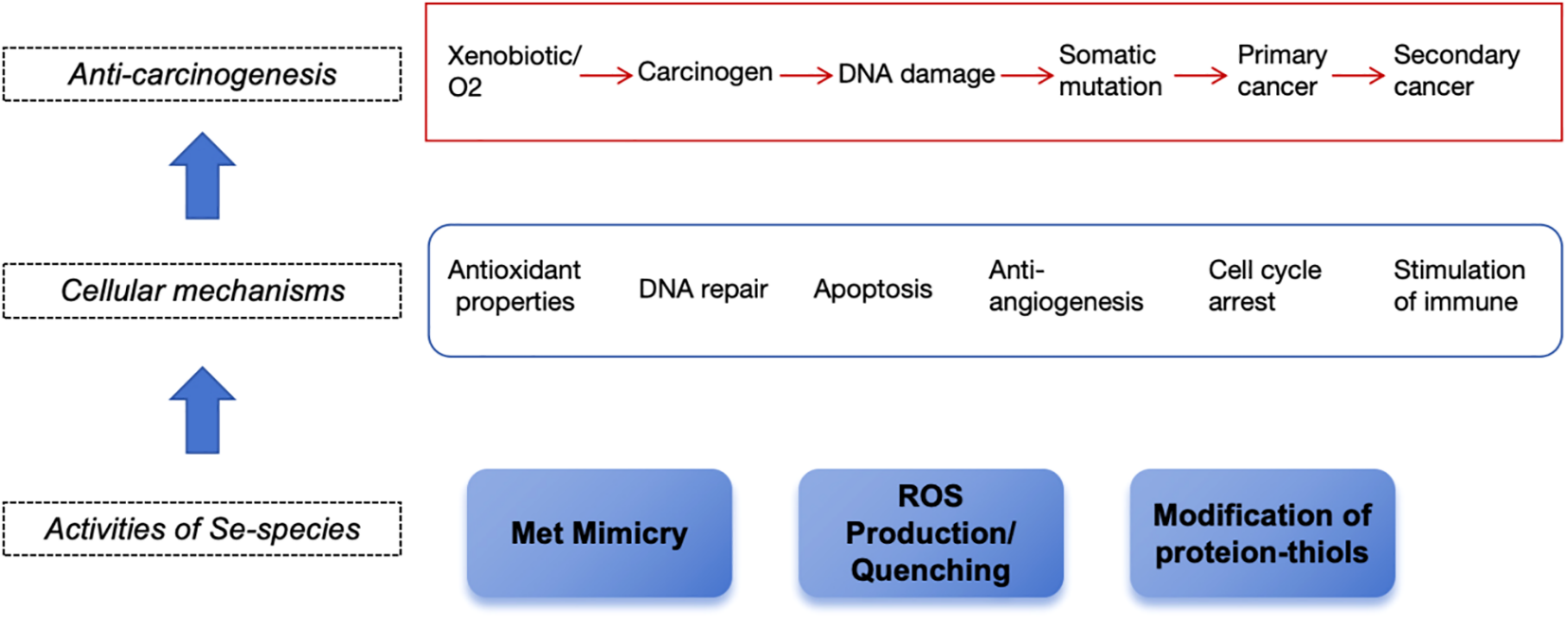
The multiple-stage action of selenium on cancer-related pathways.

Selenium primarily manifests its chemopreventive properties by ensuring proper redox equilibrium and preventing the accumulation of misfolded proteins, largely through the action of selenoproteins such as glutathione peroxidases (GPxs), thioredoxin reductases (TrxRs), and selenoprotein P, which safeguard DNA from oxidative, mutagenic stresses (121). Additional roles encompass the regulation of gene expression, the modulation of redox and hormonal metabolism, and participation in DNA repair and cellular signaling cascades. Selenoproteins function at multiple pivotal levels: they curb cell proliferation, promote apoptosis, and curtail metastasis. This is achieved through the redox modification of protein thiols and methionine mimicry in critical proteins, which can halt the cell cycle in the G1 phase (**Figure 3**). Selenoproteins that are directly or indirectly involved in maintaining redox homeostasis, including GPX, TXNRD1, SelF, and SelP, seem to influence a myriad of signaling pathways that are implicated in the initiation and advancement of cancer. Reduced selenium levels may affect the synthesis of the selenium-containing proteins mentioned above, thereby causing an imbalance in these pathways, which can lead to tumorigenesis and progression.

**Figure 4:**
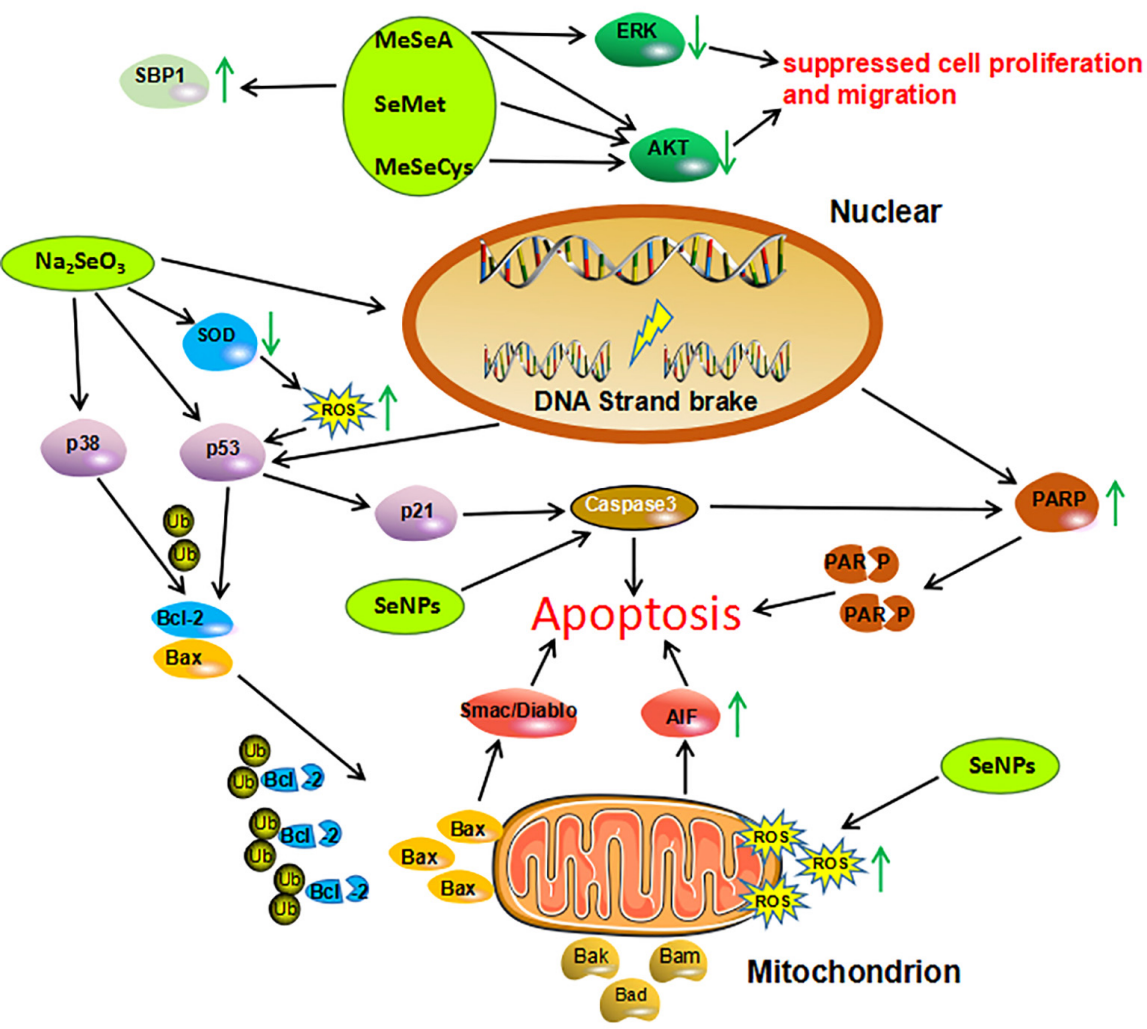
Mechanisms of action investigated in studies on Se compounds effects in cancer cells. Organic Se compounds, inorganic Se compound, Se nanoparticles are indicated in green. AIF, Apoptosis Inducing Factor; AKT, Protein Kinase B; Bad, Bcl-2 Associated Agonist of Cell Death; Bak, Bcl-2 Homologous Antagonist/killer; Bax, Bcl-2-like protein 4; Bcl-2, B-celllymphoma 2; ERK, Extracellular Regulated Kinase; MeSeA, Methylseleninic Acid; MeSeCys, Methylselenocysteine; PARP, Poly (ADP-ribose) Polymerase; ROS, Reactive Oxygen Species; SBP1, Selenium Binding Protein 1; SeMet, Selenomethione; Se-NPs, Selenium Nanoparticles; SOD1, Superoxide Dismutase 1; Ub, Ubiquitin.

Experimental studies conducted in cells and mouse models of cancer indicate that different Se compounds exert anticancer effects in cancer. Of all the tested Se compounds, the promising anticancer effects in cancer seem to be associated with Se-NPs and Na_2_SeO_3_. Laboratory evidence on specific mechanisms of action of Se in cancer is shown in **Figure 4**. According to the observations, Se compounds (both organic and inorganic compounds, as well as Se-NPs), were able in most cases to suppress cell proliferation and migration, and induce oxidative stress and apoptosis, both of which are considered to underlie antitumor effects of Se in general (122).

### 4.1 Antioxidant effects

It is a well-established fact that selenium demonstrates its anticancer capabilities primarily through its antioxidant attributes, either directly or indirectly. These attributes help maintain the cellular redox balance and shield healthy cells from oxidative damage caused by reactive oxygen species (ROS) (123). ROS are free radicals that possess unpaired electrons and are generated as a byproduct of normal biological and physiological processes. Excessive ROS levels are known to foster carcinogenesis by intensifying oxidative stress and augmenting the frequency of DNA mutations (124). Cancer cells are frequently defined by their capacity to generate and respond to elevated ROS levels. They must manage the oxidative stress conditions that are marked by heightened ROS concentrations (125). In essence, an enhanced reliance on antioxidant defenses is a defining characteristics of cancer cells.

The therapeutic potential of selenium compounds lies in their ability to modulate cellular oxidative stress, which is a significant factor in cancer treatment strategies. Selenium compounds with redox activity possess pro-oxidant characteristics that can enhance the formation of ROS. This property can be harnessed to create an oxidative environment within cancer cells, which may lead to their destruction while sparing healthy cells. Compared with normal cells, cancer cells have higher levels of ROS (126–128). Consequently, everaging these properties in combination could lead to the generation of additional reactive oxygen species, such as superoxide (O_2_^·-^) and hydrogen peroxide (H_2_O_2_), thereby throwing the oxidative homeostasis of cancer cells into even greater distress. In breast tumor cells, Se compounds react with thiols to form ROS (129). The ability of cells to generate Se metabolites more rapid oxidation of glutathione and other thiols within tumor cells, resulting in the production of ROS and peroxides. Notably, substantial disparities in thiol concentrations have been detected between malignant and normal cells. Administration of high concentrations of sodium selenite preferentially targets and eradicates cancer cells because more free radicals are produced than in normal cells and no systemic selenotoxicity is produced (130).

### 4.2 Immunomodulatory effect

The immune system stands as the body’s premier defense against the onslaught of invasive pathogens. It is tasked with the recognition and neutralization of foreign antigenic entities and works in synergy with other physiological systems to ensure the maintenance of systemic stability and equilibrium. Extensive research has been conducted to explore the potential benefits of selenium supplementation in diminishing the likelihood of cancer onset. This research suggests that selenium may function as an immunostimulant, capable of countering immunosuppression within the tumor microenvironment and bolstering anti-tumor immunity. This is achieved by stimulating the activity of immune cells, such as M1 macrophages and CD8+ T lymphocytes, and by promoting the secretion of pro-inflammatory cytokines, including interferon-gamma. On the other hand, M2 macrophages are known for their role in exerting anti-inflammatory and immunosuppressive effects, which they achieve by producing anti-inflammatory cytokines like IL-10. These cytokines play a crucial role in curbing the progression of tumors by creating an environment that is less conducive to their growth and spread (131). Selenium supplementation significantly enhances the migratory and phagocytic capabilities of macrophages that are deficient in selenium. Moreover, it facilitates a beneficial transition from a pro-inflammatory M1 phenotype to a more anti-inflammatory M2 phenotype. This shift in macrophage polarization is instrumental in dampening the pro-inflammatory responses, thereby contributing to a more balanced and regulated immune environment (132). The immunomodulatory effects of Se have been attributed primarily to the multiple activities of selenoproteins, particularly their role in maintaining redox balance (16, 133, 134). Selenoproteases such as glutathione peroxidases (GPxs) 1-4 and 6, thioredoxin reductases (TXNRDs) 1-3, methionine-R-sulfoxide reductase B1 (MSRB1), iodothyronine deiodinases (DIOs) 1-3, and selenophosphate synthetase 2 (SEPHS2) affect immune function. Adequate or excessive Se supplementation is critical for modulation of appropriate immune responses. For example, Se is incorporated into selenoenzymes that possess antioxidant properties (e.g., GPxs) to catalyze the reduction of peroxides, thus providing protection against ROS. Furthermore, other selenoproteins like TXNRD and MSRB1 are integral in regulating redox reactions and in the repair of immune cells that have been compromised by oxidative stress (116). Adequate selenium levels are vital in healthy mice for the upregulation of selenoprotein expression, as well as for the production of interferon (IFN)-γ and interleukin (IL)-6 (135). A human intervention study has demonstrated that the intake of selenium-enriched foods can lead to an increase in the levels of IL-2 and other interleukins (136). Selenium supplementation enhances spontaneous NK cytotoxicity in mouse splenocytes and specific cytotoxic T lymphocytotoxicity in peritoneal exudate cells (137).

### 4.3 Reduction of carcinogenic effects of chemical substances

Selenium can prevent the metabolic activity of certain chemical carcinogens or antagonize their metabolites thereby inhibiting the carcinogenic effects of chemical carcinogens (138). *In vitro* experiments have shown that selenium reduces the activity of hydroxylases that activate carcinogens, such as aryl hydroxylase (AHH), by more than 50% and weakens the metabolism of PAH compounds into carcinogens. FINLEY et al. found that the higher the dose of selenium added to the diet of rats, the lower the number of aberrantcrypt focus (ACF) of colon cancer in rats (139). This result also confirms that selenium can inhibit the tumorigenesis caused by chemical carcinogens mainly in the initiation and promotion stages of chemical carcinogenesis, and its related mechanism may be related to the fact that selenium reduces the activity of hydroxylase that can activate carcinogens such as aromatic amine hydroxylase in the early stage of carcinogenesis, and increases the activity of glucuronosyltransferase that can release the toxicity of carcinogens, thus blocking the activation of the metabolism of carcinogens in the organism.

An additional significant aspect of selenium’s role in cancer prevention is its chemical capacity to interact with various metals, many of which have been implicated in elevating cancer risk (140). It has been reported that selenium interacts with metals such as gold, platinum, cadmium, cobalt and mercury, which in turn can counteract the toxicity of these heavy metals (140, 141). For example, cadmium is recognized as a critical factor in the etiology of prostate and breast cancers. Selenium has been documented to offer protection against the peroxide-induced damage that is associated with cadmium exposure (142). Several of these metals have the potential to react with and inhibit vital proteins, such as thioredoxin reductase, thereby potentially exerting their toxic effects through the disruption of cellular redox balance. In such scenarios, selenium serves as a countermeasure by forming chelates with these metals, thereby mitigating their harmful impact.

### 4.4 Inducing apoptosis in cancer cells

Tumorigenesis is associated with uncontrolled apoptosis of tumor cells, and the search for highly efficient and low-toxic apoptosis-inducing agents with a clear mechanism of action is an important strategy for tumor prevention and treatment. One of mechanisms proposed for the anticancer activity of selenium is apoptosis induction which is an essential mechanism to control or eliminate the undisciplined expansion of tumors. Selenium triggers the cessation of cell proliferation and induces cell death *in vivo* through a multifaceted approach. It diminishes the expression of the cell cycle protein D1, enhances the expression of p27kip1, and activates c-Jun NH2-terminal kinase (JNK) (143). Methylselenic acid (MSeA) has been observed to augment caspase-mediated apoptosis by down-regulating the expression of survivin, Bcl-xL, and Mcl-1, which are proteins that typically inhibit apoptosis (144, 145). Furthermore, MSeA instigates a cell cycle arrest at the G1 phase, a critical checkpoint in the cell cycle, which is correlated with an upregulation in the expression of p27kip1 and p21cip1, both of which are cyclin-dependent kinase inhibitors that play a crucial role in regulating cell cycle progression (146). The combination of selenocysteine and 5-fluorouracil can regulate the intracellular redox system, induce cellular oxidative stress and DNA damage, and block the extracellular signal-regulated kinase (ERK) signaling pathway, thus promoting 5-fluorouracil-induced apoptosis of tumor cells, and enhancing its antitumor effects (147). Sodium selenite, the most abundant inorganic selenium compound in nature, reduces mitochondrial membrane potential and enhances the antiproliferative and apoptosis-inducing effects of polyene paclitaxel on prostate cancer cell PC3 (111). The synthesized selenium compound diphenylselenocyanate can induce DNA damage through ROS, upregulate p53 gene expression, activate the caspase signaling pathway, thereby increasing the sensitivity of Ehrlich ascites cancer cells to cyclophosphamide (148). In addition, it was found that nanosized selenium significantly enhanced the killing effect of irinotecan hydrochloride on HCT-8 cells through p53-mediated apoptosis pathway (149). The above studies also suggest that ROS generation, upregulation of p53, and reduction of mitochondrial membrane potential play important roles in selenium-assisted anti-tumor processes.

### 4.5 Inhibition of tumor angiogenesis and tumor metastasis

Tumor angiogenesis is a pivotal process that enables tumor cells to proliferate, infiltrate surrounding tissues, and disseminate to distant sites. Angiogenesis and tumor progression are closely related and can affect the tumor microenvironment as well as the sensitivity of tumor cells to chemotherapeutic agents or radiotherapy (150). The suppression of tumor invasion and metastasis constitutes a significant mechanism underlying the anti-metastatic capabilities of selenium in cancer therapy. Hypoxia-inducible factor-1α (HIF-1α) serves as a critical transcription factor that orchestrates cellular responses to hypoxic conditions. Its activity is instrumental in sustaining the energy metabolism of cancer cells, fostering neovascularization, and enhancing both the proliferation and migration of tumor cells. *In vitro* investigations have demonstrated that selenomethyl selenocysteine is capable of diminishing ROS levels, stabilizing the levels of prolyl hydroxylase-2 (PHD2) and prolyl hydroxylase-3 (PHD3), and downregulating HIF-1α expression in colon and head and neck cancer cells. These findings suggest that selenomethyl selenocysteine may enhance the responsiveness of tumor tissues to anticancer agents by reducing HIF-1α expression in mice bearing tumors, thereby potentially improving the efficacy of cancer treatments (151). Liu et al. showed that selenomethyl selenocysteine, sodium selenite and methyl selenite could enhance the inhibitory effect of cyclophosphamide on canine breast cancer cells by down-regulating the expression of VEGF, angiotensin II (Ang II) and HIF-1α, which are related to the growth and vascularity of tumors, and by up-regulating the expression of phosphatase and tensin homologous genes (152). Matrix metalloproteinases (MMP) -2 and MMP-9 are enzymes that break down the extracellular matrix and basement membrane, which are crucial barriers preventing the spread of cancer cells. These enzymes play a pivotal role in facilitating tumor invasion and metastasis by enabling cancer cells to penetrate and migrate through these protective layers. The urokinase-type plasminogen activator (uPA) system is another factor that is closely linked to tumor progression. It is implicated in the processes of tumor invasion and metastasis, and its elevated activity is often correlated with a reduction in patient survival times, highlighting its significance as a prognostic indicator in cancer (153). Selenite impedes the invasive capabilities of cancer cells by curtailing the activity of MMP-2 and MMP-9, as well as uPA (154). Another experiment showed that MSeH acts as a nutritional adjuvant to reduce melanoma cell metastasis by inhibiting integrin expression and MMP (155). Ultimately, Se forestalls the invasive and metastatic progression of tumors by suppressing the activity of MMPs. These enzymes are instrumental in the breakdown of the extracellular matrix (ECM), the penetration of basement membranes, the processes of endocytosis and exocytosis, and the promotion of further angiogenesis. Moreover, there is a necessity for additional animal and human intervention studies to ascertain the efficacy of selenium compounds in chemoprevention strategies and their potential to obstruct the metastatic cascade of cancer.

### 4.6 Inhibition of tumor cell cycle progression

Selenoprotein H plays a crucial role in modulating cell cycle progression and in curbing the onset of rampant cell proliferation. The intricate mechanisms through which selenium influences the inception and advancement of cancer could potentially be linked to the function of selenoprotein H. This selenoprotein’s involvement in maintaining cellular homeostasis may be a critical factor in selenium’s chemopreventive properties, particularly in the context of cancer development. Knockout studies in human colorectal cancer cells by BERTZ et al. found that selenoprotein H knockout cells showed faster cell cycle transitions, and high levels of selenoprotein H significantly inhibited cell proliferation and the G1/S phase of cell mitosis (156). CHIGBROW et al. found that the mechanism of selenium inhibition of tumor cell cycle progression may be related to selenium regulation of RNA expression of mitotic cell cycle protein B and cdc2 kinase activity by analyzing the cell cycle effects of selenoethanethionine in colon cancer cells in their laboratory (157). Thus, selenium inhibition of tumor cell cycle progression may result from multiple mechanisms.

### 4.7 Others

#### 4.7.1 Protecting the structure and function of genetic material

Selenium is also capable of stimulating DNA repair pathways, which are essential for the removal of DNA damage. Considering the vital function of selenoproteins, such as glutathione peroxidase and thioredoxin reductase, in providing antioxidant protection and preserving a reduced intracellular milieu, selenium plays a pivotal role in expediting the DNA damage repair process. This acceleration is achieved by boosting the synthesis of selenoproteins, which in turn reinforces the cell’s capacity to counteract oxidative stress and maintain genomic integrity (158). SeM enhances p53 activity and protects cells from DNA damage through its antioxidant activity (159). A study involving wild-type (WT) and p53 knockout (p53 -/-) mouse embryonic fibroblasts (MEFs) demonstrated that pretreatment with 10 μmol/L of selenomethionine (SLM) for 15 hours, followed by exposure to cisplatin or oxaliplatin, revealed a protective effect of selenium on WT-MEFs. This protection was manifested by a reduction in DNA damage, highlighting the role of selenium in safeguarding cells from the genotoxic effects of these platinum-based chemotherapeutic agents (160).

#### 4.7.2 Influencing the expression of oncogenes and oncogenes

Recent studies have confirmed that selenium can affect the expression of oncogenes and oncogenes in the body. ANVAR et al. carried out an *in vitro* study to investigate the impact of selenium on the telomerase activity in human umbilical cord mesenchymal stem cells (161). The findings indicated that following supplementation with sodium selenite, there was a marked decrease in the expression levels of the oncogene c-myc, accompanied by a significant elevation in the expression levels of the tumor suppressor gene p53. The activity of telomerase was inhibited to prevent the occurrence and development of tumors and the proliferation of cancer cells.

#### 4.7.3 Involving in DNA repair and cellular senescence

Selenium’s capacity to stimulate DNA damage repair is a significant aspect of its anticancer activity. Recognizing the essential function of selenoproteins, such as glutathione peroxidase and thioredoxin reductase, in antioxidant defense and in preserving a reduced intracellular state, selenium is able to expedite the DNA repair process. This acceleration is achieved by augmenting the synthesis of selenoproteins, which in turn bolsters the cell’s ability to counteract oxidative stress and to safeguard the integrity of its genetic material (158). It was found that the treatment of leukocytes with bleomycin induced DNA damage, and the addition of selenomethionine significantly reduced bleomycin-induced DNA breaks and improved the repair of DNA damage (162). Cellular senescence is an important step in suppressing tumorigenesis. Selenium compounds were able to activate DNA damage response-associated kinases and caused senescence in human embryonic lung fibroblasts cell 5 (MRC-5), but not in human colorectal cell 116 (HCT-116) and prostate cancer cells 3 (PC-3) (163). Deletion of phosphatase Pten protein is often detected in specimens of human prostate cancer, and the effect of selenium was examined by using mice lacking this gene, it was found that a large number of senescent cells could be detected in the defective parts of the prostate gland after 4 weeks of continuous treatment with methyl selenate, indicating that selenium-induced cellular senescence can indeed inhibit tumorigenesis (164).

## 5 Forms of selenium action in tumor supression: selenoproteins, selenium compounds, selenium nanoparticlels

### 5.1 Selenoproteins

Selenium exerts its biological influence primarily through the action of selenoproteins (165). In these selenoproteins, the Selenocysteine (Sec) residue is often observed at their enzymatic active site, which is critical for their function and activity (166). To date, a catalog of 25 selenoprotein-encoding genes has been delineated in the human genome (**Table 1**). These selenoproteins are ubiquitously present across a spectrum of organs and tissues, each characterized by distinct substrate affinities and functional roles (167–169).

**Table 1:** Selenoproteins and their functions in the human body.

Selenoprotein	Abbreviation	Function	Sec location	Protein size
Glutathione peroxidase 1	GPx1	Metabolize hydrogen peroxide and some organic hydroperoxides	47	201
Glutathione peroxidase 2	GPx2	Antioxidant activity in gastroin testinal tissues	40	190
Glutathione peroxidase 3	GPx3	Reduce H2O2, fatty acid hydroperoxides, and phospholipid hydroperoxides in the plasma and thyrocytes	73	226
Glutathione peroxidase 4	GPx4	Reduce phospholipid- and cholesterol-hydroperoxides by using GSH	73	197
Glutathione peroxidase 6	GPx6	Reduce olfactory organs H2O2	73	221
Thioredoxin reductase 1	TrxR1	Antioxidant activity and regenerate reduction of thioredoxin	498	499
Thioredoxin reductase 2	TrxR2	Regenerates reduced thioredoxin in mitochondria	655	656
Thioredoxin reductase 3	TrxR3	Redox regulation	522	523
Iodothyronine deodinase 1	DIO1	Production of T3 in thyroid and peripheral tissues	126	249
Iodothyronine deodinase 2	DIO2	Production of T3 in peripheral tissues	133, 266	273
Iodothyronine deodinase 3	DIO3	Inactivates thyroid hormone	144	278
Methionine-R-sulfoxide reductase	MSRB1	Restores oxidatively damaged methionine (Met-sulfoxide) to native configurations	95	116
Selenophosphate synthetase 2	SEPHS2	Sec synthesis	60	448
Selenoprotein F	SelF	Oxidoreductase that may assist in disulfide formation and protein folding/Correcting misglycosylated	93	162
Selenoprotein H	SelH	Cell cycle regulation & cancer prevention	38	116
Selenoprotein I	SelI	Phospholipid biosynthesis	387	397
Selenoprotein K	SelK	Antioxidant activity/immunity/inflammation	92	94
Selenoprotein M	SelM	Maintenance of Ca2+ ions/Antioxidant activity	48	145
Selenoprotein N	SelN	Growth and development of muscles	428	556
Selenoprotein O	SelO	Regulation of redox reactions	667	669
Selenoprotein P	SelP	Transportation of Se to brain and other tissues of body	59, 300, 318, 330, 345, 352, 367, 369, 376, 378	381
Selenoprotein S	SelS	Deletes the misfolded proteins in endoplasmic reticulum and responds to endoplasmic reticulum stress	188	189
Selenoprotein T	SelT	Regulation of endocrine secretion/Ca2+ mobilization	36	182
Selenoprotein V	SelV	Expression of taste/Regulation of redox reactions	273	346
Selenoprotein W	SelW	Oxidative stress regulation, Bone remolding	48	145

Research has demonstrated that selenoproteins, such as selenoprotein P (SelP), glutathione peroxidase (GPx), thioredoxin reductase (TXNRD), and selenoprotein F (SEP15, SelF), exert regulatory effects on tumorigenesis and tumor progression through modulation of cancer-associated signaling pathways (170). The correlation between single nucleotide polymorphisms (SNPs) within selenoprotein genes and cancer susceptibility has been a subject of investigation, encompassing genes like SelP and GPx, as well as TXNRD, selenoprotein N (SEPN1, SelN), selenoprotein S (VIMP, SelS), and selenoprotein W (SEPW1, SelW) (171, 172). Since selenium is of great significance in cancer and immune system function, it is necessary to further study the role of selenoproteins in cancer development, growth and progression. At present, most of the studies on the relationship between selenium and cancer are observational studies. Because there are still many conflicting conclusions in the related research, further research is needed to clarify the correlation between selenium and cancer. This comprehensive review delineates a selection of pivotal and extensively investigated selenoproteins, aiming to inspire innovative directions for forthcoming scientific inquiries.

#### 5.1.1 Glutathione peroxidase

The glutathione peroxidase (GPx) family is a group of the most well characterized selenoproteins, and it holds the distinction of being the inaugural class of selenium-enriched enzymes to be discovered, marking a significant milestone in the understanding of selenium’s biochemical role (173, 174). The antioxidant power of GPxs is well documented, and they typically use glutathione primarily as an electron donor to catalyze the reduction of peroxides, which in turn helps the body defend itself against free radicals. The glutathione peroxidase family, which has been found to include eight members of GPx1-8, is one of the most intensively studied SePs to date in terms of its contribution to tumourigenesis. As of now, the scientific community has identified five selenium-dependent GPx, which include GPx1, GPx2, GPx3, GPx4, and GPx6. These enzymes are distinguished by their reliance on selenium for their catalytic activity. In contrast, GPx5, GPx7, and GPx8 are notable for incorporating Cys in place of Sec, highlighting the diversity within this family of enzymes (42, 175). The GPx play a pivotal physiological role in safeguarding cells and tissues against the onslaught of oxidative stress, particularly from hydroperoxides. Among them, GPx1 is one of the most abundantly present and widely expressed selenoproteins (176). Several studies have shown that GPx1 is associated with cancer when its expression is inhibited or reduced in particular (177, 178). Notably, an overabundance of GPx1 has been demonstrated to shield cancer cells from the potent oxidizing effects of anticancer therapeutics. Furthermore, the levels of GPx1 have been observed to exhibit a direct correlation with the progression to advanced metastatic cancer, underscoring its potential role in cancer biology (179, 180). Among the critical antioxidant enzymes in humans, GPx4 holds a significant position. It plays a crucial role in mitigating the process of non-apoptotic cell death, colloquially termed “ferroptosis,” by counteracting the aggregation of lipid ROS and the subsequent occurrence of lipid peroxidation under oxidative stress conditions (181). In such a situation, the ablation of GPx4 has been demonstrated to render tumor cells more susceptible to ferroptosis-eliciting agents, suggesting that GPx4 may emerge as a promising target for the development of novel therapeutic interventions (181, 182).

#### 5.1.2 Thioredoxin reductase

Thioredoxin reductase (TrxR) is a homodimeric enzyme that boasts a sophisticated architecture, featuring a flavin adenine dinucleotide (FAD) and a reduced nicotinamide adenine dinucleotide phosphate (NADPH) binding site for each of its monomers, thereby facilitating its essential role in cellular redox homeostasis (183). The primary function of TrxR is to reduce thioredoxin, which is used in a variety of processes (184). As normal cells become malignant, the number of TrxR system components increases dramatically. For instance, Thioredoxin reductase 1 (TrxR1) is meticulously governed by the nuclear factor erythroid 2-related factor 2 (Nrf-2), a pivotal transcription factor that modulates its expression. Notably, TrxR1 exhibits elevated levels of expression across a spectrum of malignancies, encompassing hematological neoplasms such as lymphoma and multiple myeloma, underscoring its potential as a therapeutic target in oncology (185). Overexpression of thioredoxin reductase 2 (TrxR2) in cancer cells is often considered a critical factor in the complex interplay of tumorigenesis, disease progression, and the resistance to apoptosis, underscoring its significant role in the intricate dynamics of cancer development and survival. The suppression of thioredoxin reductase 2 (TrxR2) results in a marked elevation of ROS within the mitochondrial compartment. This occurs through the impairment of thioredoxin 2 (Trx2) activity, which subsequently triggers the release of a cascade of proapoptotic factors, including the notable cyclophilin D, thereby influencing the apoptotic pathways and cellular homeostasis. Therefore, selective inhibition of TrxR2 has been paid much attention as a strategy to kill cancer cells and induce apoptosis recently (186). Studies have shown that increased TrxR enhances oncogenic processes in a number of ways, including enhancing tumor formation, inducing angiogenesis, and increasing resistance to cancer therapies (187). Cancer cells typically exhibit a profound dependence on aerobic glycolysis as their primary energy-generating pathway (188). In addition, in the pentose phosphate pathway (PPP), more glucose is required to produce reducing equivalents. Tumor cells harness this metabolic pathway to meticulously preserve redox equilibrium, which is essential for sustaining their viability, proliferation, and the perpetuation of cell division, thereby facilitating their relentless growth and expansion (189, 190). In brief, inhibition of TrxR augments the susceptibility of tumor cells to oxidative stress, bolsters the selective eradication of cancerous cells, and instigates the cascade of apoptosis, which has received widespread attention in recent years making TrxR a potential target for cancer therapy (191, 192).

#### 5.1.3 Selenoprotein P

Selenoprotein P (SelP) stands out as an exceptional member within the selenoprotein family due to its distinctive composition; SelP can harbor up to ten Sec residues, a feature that contrasts with the majority of selenium-enriched proteins, which typically incorporate only a single Sec residue. This abundance of selenium-laden residues strongly implies a pivotal function in the transport and distribution of selenium throughout the body, underscoring the unique biological role of SelP in selenium transport. Apart from GPx3, SelP is among the select group of secreted selenoproteins predominantly synthesized in the liver, which serves as a principal hub for selenium metabolism (193). During the inflammatory response, specifically the acute phase reaction, SelP biosynthesis is reduced, which disrupts selenium transport and selenium metabolism, which may result in lower selenium levels in cancer patients (194). Furthermore, the presence of single nucleotide polymorphisms (SNPs) within the human SelP gene can result in an altered response to dietary selenium intake. Such genetic variations may restrict the efficiency of selenium transport, consequently diminishing the activity of selenium-dependent enzymes. This reduction in enzymatic activity could potentially elevate the risk of developing cancer, highlighting the intricate relationship between genetics, selenium metabolism, and cancer susceptibility (195, 196). Overall, extensive research has conclusively shown that levels of SelP are consistently diminished across a multitude of cancer types. This decrease in SelP expression exhibits a negative correlation with the progression of the disease. Notable examples include hepatocellular carcinoma, cancers of the gallbladder and biliary tract, gastric adenocarcinoma, colorectal cancer, and prostate cancer. These findings underscore the potential of SelP as a biomarker for disease severity and its pivotal role in the pathogenesis of various malignancies (118, 197–200).

#### 5.1.4 Selenoprotein F (the 15 kDa selenoprotein)

Selenoprotein F (SelF) is a 15kDa thioredoxin-like oxidoreductase localized in the endoplasmic reticulum (ER), containing a quintessential thioredoxin-like fold and having a Cys-X-X-Sec motif, suggesting that they have the redox activity of thiol disulfide bond oxidoreductases (201, 202). SelF was first identified in human T cells, yet its expression is not confined to these immune cells. It is also abundantly present in a range of epithelial tissues, including the liver and prostate (203). Moreover, SelF was found to be highly expressed in normal liver and prostate tissues, but reduced in the malignant counterparts of these organs (204). The impact of Selenoprotein F expression on tumorigenesis has been a subject of investigation across various cancer types. Research has consistently documented a decrease in SelF levels within tumor cells and cell lines derived from tumors. Intriguingly, these studies converge on the finding that the downregulation of SelF mitigates the pro-tumorigenic characteristics, implying a protective role for this selenoprotein in the context of cancer development. Down-regulation of SelF in cancer cell lines leads to up-regulation of p21 and p27 (cell cycle inhibitors) and subsequently induces a slowdown in cellular proliferation and triggers a state of growth arrest specifically in colon and liver cancer cells (205–207). Knockdown of the 15 kDa Selenoprotein in HT-29 and HCT116 human colon cancer cell lines effectively halted both anchorage-dependent and anchorage-independent cell proliferation, which was achieved through the induction of a G0/G1 cell cycle arrest (207). Similarly, Tsuji et al. noted that the ablation of Selenoprotein F effectively thwarted the emergence of aberrant crypt foci, which are early indicators of colonic neoplasia. Concurrently, this deletion led to the activation of IFN-γ and the subsequent induction of gene expression regulated by the signal transducer and activator of transcription 1 (STAT1) following the administration of azoxymethane (AOM), a potent carcinogen (208). Overall, these results presented thus far exhibit a degree of inconsistency, which suggests that further investigation is imperative. Additional research will be crucial in elucidating the comprehensive role of Selenoprotein F across various cancer types, aiming to reconcile the seemingly contradictory data and to establish a more definitive understanding of its impact on oncogenesis.

### 5.2 Selenium compounds

**Figure 5:**
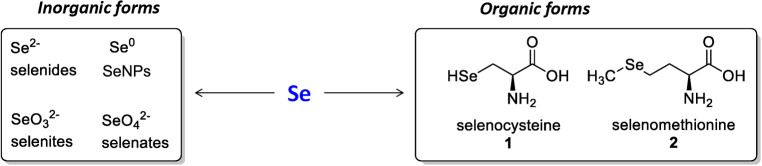
The most important inorganic and organic forms of selenium.

Selenium compounds include inorganic selenium compounds and organic selenium compounds, with the main organic forms being selenomethionine and selenocysteine, and the inorganic forms being selenite, selenide, selenate, and selenium, which are integral to various biological functions (**Figure 5**) (209).

#### 5.2.1 Inorganic Se compounds

Selenium exhibits a range of four natural valence states: elemental selenium (0, SeNPs), selenide (-2; Se^2-^), selenite (+4; SeO_3_^2-^) and selenate (+6; SeO_4_^2-^). The biological functionality and potential toxicity of inorganic selenium compounds are significantly influenced by these valence states. Common inorganic selenium compounds are hydrogen selenide (H_2_Se), hydrogen metal selenide (HSeM), dimetallic selenide (M_2_Se), hydrogen diselenide (H_2_Se_2_), metal diselenide (M_2_Se_2_), metal selenium cyanate (MSeCN) and metal selenium sulfate (SO_3_SeM_2_).

Selenite stands as the most extensively researched form of inorganic selenium compounds, renowned for its remarkable chemopreventive and anticancer properties. Selenite is effective in inhibiting the proliferation of a wide range of cancer cells, and it has been shown to exert anticancer activity in a variety of cancer cell lines, including prostate, breast, lung, liver, bladder and osteosarcoma (210). In experiments using selenite to test different human cancer cell lines, it was found that lung cancer cells may be more susceptible to the chemopreventive and anticancer actions of selenite (211–213). Sodium selenite (Na_2_SeO_3_) has received approval from the United States Food and Drug Administration (FDA) for use as a dietary supplement in animal nutrition. It was found that Na_2_SeO_3_, when applied to cholangiocarcinoma cells, significantly reduced invasion, migration and epithelial-mesenchymal transition by causing apoptosis and down-regulation of N-calmodulin (214). In some cases, selenate has demonstrated superior efficacy over selenite in both the prevention and treatment of cancer. This distinction in effectiveness can be observed even within the same cancer type, highlighting the potential variability in response among different cell lines (215, 216).

#### 5.2.2 Organic Se compounds

Organic selenium compounds have attracted a lot of attention in the field of cancer research, mainly because they are usually more bioavailable and less toxic compared to inorganic selenium compounds (217). Organic selenium compounds constitute a diverse array of molecules that can be categorized into distinct families based on their functional chemical structures: Selenides/diselenides, selenocyanates, selenoamino acid derivatives (e.g., SeMet and MSC), methyl selenoic acid (MSA; CH_3_SeO_2_H), selenoheterocyclic compounds, and a variety of other selenium-containing compounds. To date, there have been numerous scientific studies on organic selenium compounds, many of which have examined their role in cancer prevention and treatment. These organoselenium compounds demonstrate potent anticancer and chemopreventive properties by engaging a multitude of action mechanisms including reduction of oxidative stress, induction of apoptosis, and enhancement of chemotherapeutic drug activity (218–221). Research has consistently illustrated that selenium amino acid derivatives, such as selenomethionine (SeMet) and methylselenocysteine (MSC), are capable of stimulating apoptosis in a broad spectrum of human solid tumors. This pro-apoptotic effect is a significant mechanism in the fight against cancer, as it targets the elimination of malignant cells. Moreover, MSC has been found to offer supplementary safeguarding against the adverse effects of anticancer therapeutics, potentially reducing the toxicity associated with these treatments (222–224). MSA is the oxidized form of methylselenol (CH_3_Se-) converted from selenoamino acids (e.g. SeMet and MSC) (225). Multiple studies have proven that MSA is an excellent anticancer agent against a variety of cancer models, including lung cancer, breast cancer, melanoma, and especially prostate cancer (226–228). MSA exerts its anticancer effects through various mechanisms; it effectively curbs the uncontrolled expansion of cancer cells by instigating the apoptotic cascade (229), blocking the cell cycle (230) and anti-angiogenic activity (231). During the study, MSA was also found to enhance the efficacy of several chemotherapeutic agents, *i.e.*, paclitaxel (232), TAM (233), adriamycin, cytarabine cytarabine (234) and cyclophosphamide (152). Based on current evidence, MSA may be considered a promising drug candidate for cancer treatment.

The class of selenium compounds encompasses a diverse array of substances, including selenocyanates and selenium-embedded heterocyclic compounds, which have garnered attention for their promising chemopreventive and anticancer attributes. This group extends from the extensively researched p-xylene selenocyanates and benzyl selenocyanates to the more recently discovered, innovatively active compounds. These novel entities ingeniously fuse the selenocyanate moiety with a variety of heterocyclic rings, quinones, or steroidal frameworks, thereby expanding the horizons of selenium-based therapeutics. Heterocyclic organoselenium compounds, exemplified by ibiselenocyanine and ethylselenocyanine (also recognized as BBSKE), represent a category of diminutive molecules that harbor significant potential in the realm of cancer therapy (235, 236).

The attributes of selenium compounds herald a new era of optimism within the domain of oncology, particularly in enhancing the resilience of tumors against conventional chemotherapy and radiotherapy, as well as mitigating the profound side effects that often accompany these treatments. The proposition of employing selenium compounds as novel, prospective pharmacological agents or adjuvants in cancer therapeutics is substantiated by their reduced toxicity, heightened selectivity and efficacy, and the prospect of attenuating the adverse effects typically linked to mainstream anticancer interventions. In summation, while the potential of these selenium-enriched compounds is undeniably promising, there remains an imperative for further comprehensive investigation, particularly *in vivo* studies, to ascertain the consequences of their prolonged consumption and to evaluate the enduring impacts related to their sustained application.

### 5.3 Selenium nanoparticles

Selenium nanoparticles (SeNPs) have received extensive attention in the biomedical field due to their unique physical, chemical and biological properties. SeNPs exhibit many advantages over conventional organic and inorganic selenium compounds, including low toxicity, anticancer and antimicrobial activities. SeNPs can also act as immunomodulators to inhibit tumor growth by enhancing anti-tumor immunity, such as modulating tumor-associated macrophages and activating specific T lymphocytes. In recent years, there has been a surge of interest in the development of nanomaterials that possess augmented anticancer capabilities while minimizing adverse effects on the human body, positioning them as promising candidates for cancer treatment. In this context, nanoparticles enriched with selenium are under rigorous investigation for their potential as cancer therapeutics. This interest is fueled by the fact that selenium is an indispensable trace element, and selenium-laden nanomaterials exhibit enhanced biocompatibility. These characteristics make selenium-containing nanoparticles an attractive avenue for research, with the potential to revolutionize cancer treatment by offering targeted, effective therapies with reduced toxicity.

Selenium nanoparticles are effective against a wide range of cancers, including colon, liver, breast, prostate and lung cancers. In 2010, a pioneering study by Shakibaie and colleagues revealed that SeNPs exerted a toxic effect on the fibrosarcoma cell line HT-1080. Remarkably, these nanoparticles were found to impede the invasive and metastatic potential of cancer cells by modulating the expression levels of MMP-2, a key enzyme involved in the degradation of extracellular matrix components and a facilitator of cancer cell dissemination (237). In a significant study, Kong and colleagues delved into the cytotoxic effects of SeNPs on the prostate cancer cell line LNCaP, and they found that SeNPs effectively curbed the proliferation of these cancer cells by triggering a cascade of caspase-mediated apoptosis (238). Toubhans and colleagues conducted a compelling study that illustrated the profound effects of inorganic selenium nanoparticles on ovarian cancer cells. They demonstrated that these nanoparticles elicited nanomechanical responses, including alterations in cell surface roughness and membrane rigidity, ultimately leading to apoptosis in SKOV-3 and OVCAR-3 ovarian cancer cell lines (239). Zhai et al. found that SeNPs stabilized with chitosan (CS) of different molecular weights exhibited significantly reduced cytotoxicity in BABLC-3T3 and Caco-2 cells (53). In addition, SeNPs can be synergistically used with anticancer drugs for cancer therapy, thereby enhancing anticancer activity and minimizing toxic effects. Yang et al. found that adriamycin alone destroyed 20% of cancer cells, while SeNPs combined with adriamycin destroyed more than 50% of cancer cells (240). During breast cancer treatment, anastrozole is used to inhibit the growth of the enzyme aromatase, which causes side effects such as bone fractures and osteoporosis (241, 242). These can be prevented by combining anastrozole with SeNPs.

**Figure 6:**
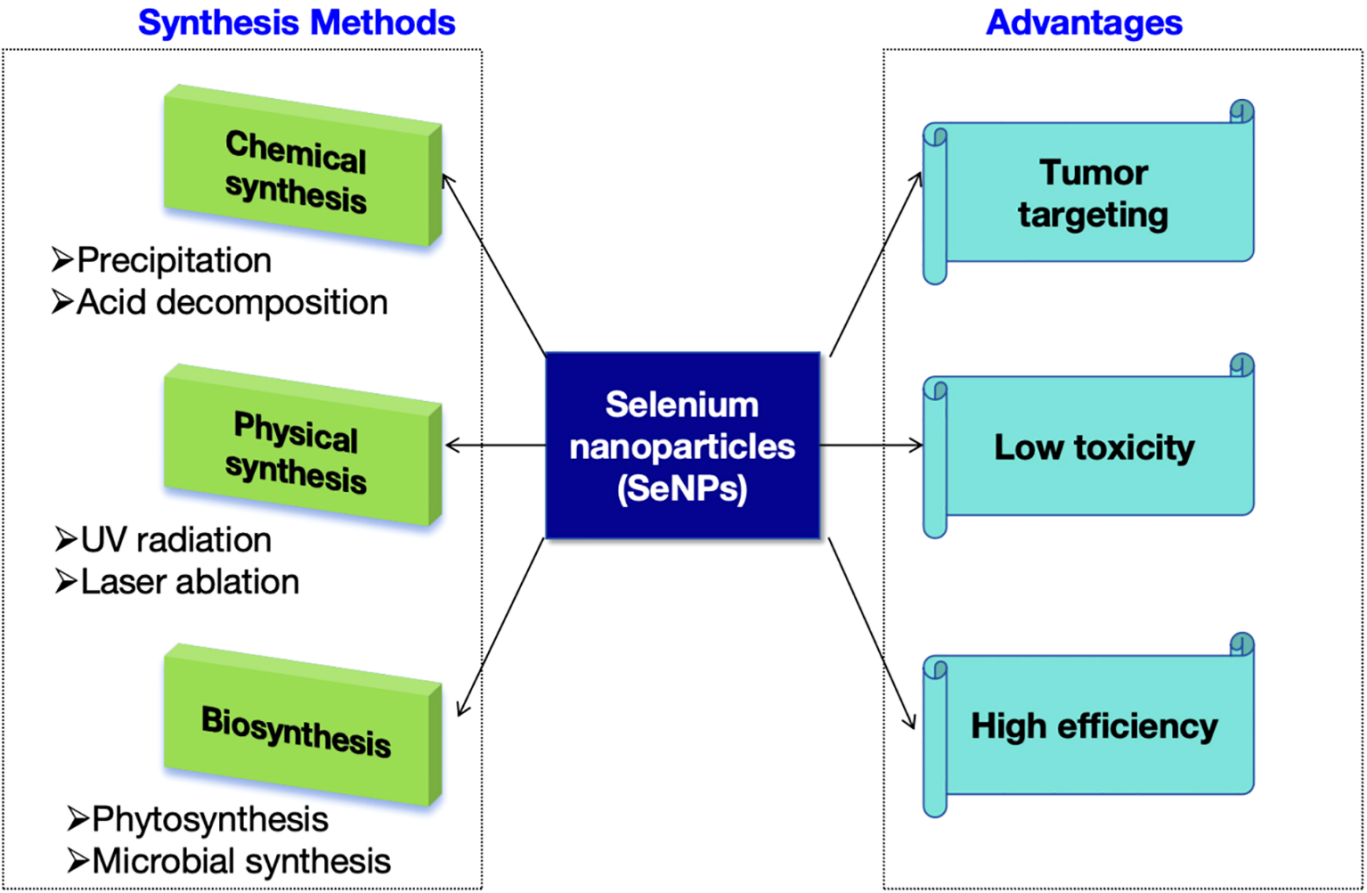
The synthesis methods and advantages of selenium nanoparticles.

Currently, several methods have been proposed to prepare SeNPs, which are usually categorized into three main groups based on different production principles: chemical synthesis (precipitation, acid decomposition, *etc.*), biosynthesis (photosynthesis, microbial synthesis, *etc.*), and physical synthesis (UV radiation, laser ablation, *etc.*) *(* 243). Among them, chemical synthesis is considered to be the most common method for the preparation of SeNPs. In chemical synthesis, Se in the +4 valence state (e.g., selenite, selenite, or SeO_2_) is commonly used as a precursor, while reducing agents (e.g., ascorbic acid and glutathione [GSH]) and stabilizers (e.g., chitosan and pectin) are used for the formation and maintenance of SeNPs (53, 244, 245). To maximize their efficacy in cancer therapeutics and prophylaxis, chemically synthesized SeNPs are frequently tailored with particular molecules that bestow advantageous attributes for real-world utilization. For instance, the conjugation of SeNPs with additional biologically active compounds can significantly amplify their therapeutic impact on specific cancer subtypes, surpassing the performance of unmodified SeNPs (246, 247). In addition to direct therapeutic effects, chemically modified SeNPs can also serve as carriers, conferring favorable properties to the carriers, such as tumor targeting (248), high efficiency (249) and low toxicity (250). Overall, the chemical synthesis of SeNPs stands as the predominant technique for their acquisition and modification. This method is favored due to its simplicity and the ease with which the process can be managed and fine-tuned. Nevertheless, it is imperative to take into account the potential for environmental contamination and the accumulation of these chemically synthesized materials within the body. Compared to chemically synthesized SeNPs, biosynthesized SeNPs appear to be more environmentally friendly and biologically safe (**Figure 6**). Therefore, there is a growing interest in biosynthesized SeNPs, also due in part to their extraordinary biocompatibility, sustainability and affordability (251). These selenium nanoparticle-enriched biomaterials are ingeniously crafted either extracellularly or intracellularly by a diverse array of organisms, including selective plants, bacteria, fungi, and other biological entities (252–254).

Despite the large number of studies reporting positive biomedical effects, there are still safety and toxicity issues for the clinical use of selenium. Although the safe range of selenium is narrow, the safe range of selenium is not only related to its concentration but also to the form in which it is present. For example, it has been shown that exceeding the upper limit of selenium intake (400 μg/d) can lead to selenotoxicity, but no significant symptoms of toxicity were observed when selenium was ingested in the form of SeNPs (1,600 μg/d), and some selenotoxicity symptoms did not occur until even an intake of 3,200 μg/d (255).

SeNPs have lower toxicity and better biological functions than organic selenium such as selenomethionine (SeMet), selenocystine (Sec) and seleno-methylselenocysteine (SeMetSec). Sec and seleno-methylselenocysteine have lower toxicity and better biological functions. For instance, SeMet significantly increased alanine aminotransferase, aspartate transferase, and lactate dehydrogenase levels in the liver for prolonged periods relative to SeNPs, exhibiting acute liver injury (256). The LD50s for SeNPs, SeMet and SeMetSec were 92.1, 25.6 and 14.6 mg/kg, respectively, making SeNPs less toxic than organic selenium. On the other hand, SeNPs have lower toxicity and better biological functions than inorganic selenium (selenite and selenium dioxide). For example, inorganic selenium significantly reduced glutathione reductase levels in the liver and increased the production of lipid peroxides in the liver compared to SeNPs, thereby reducing the activity of the antioxidant enzymes SOD and CAT in the liver (257). The acute toxicity of SeNPs is about seven times that of sodium selenite: their LD50s are 113 and 16 mg/kg, respectively. *In vitro*, SeNPs reacted with glutathione at a rate 1/10 that of sodium selenite. *In vivo* studies in rats have also shown that SeNPs have similar efficacy and lower acute toxicity compared to sodium selenite. In addition, selenium dioxide (LC50 = 6.7 mg/L) had a lower LC50 relative to SeNPs (LC50 = 41.0 mg/L). The high toxicity of inorganic selenium (selenite and selenium dioxide) and organic selenium is related to their ability to oxidize sulfhydryl groups, leading to inactivation of sulfhydryl-containing enzymes. Thus, compared to other forms of selenium, SeNPs have a wider safety profile and can be used as potential chemotherapeutic agents with lower toxicity risks.

## 6 Conclusion

Global cancer statistics show an alarming number of patients suffering from the disease. Cancer places a heavy economic burden on citizens. Society is under increasing economic pressure from cancer due to the high cost of cancer treatment and diagnosis (258). Despite the heavy economic burden, cancer remains a complex global problem, and stands as the second leading cause of mortality in the United States, underscoring the complexity and pervasiveness of this disease (259). Possible preventive measures are therefore being sought to prevent the development of cancer and thus holds the promise of curtailing the incidence of new cases and deaths. To date, many drugs have been introduced for cancer treatment in different cancer stages. However, some of these drugs are less selective for cancer cells and can be toxic to healthy cells.

For decades, selenium has been considered an essential trace element that exerts its biological functions of antioxidant defence, redox signalling and immune response through various selenoproteins. Although some clinical trials have shown no significant benefit of selenium in preventing cancer, a large body of evidence suggests that selenium is an effective anticancer agent in some cases. Selenium boasts a well-established history as a potent agent in the prevention of cancer. A multitude of factors that influence the salutary effects of Se compounds have been pinpointed, including the baseline Se status, the dosage levels administered and the specific forms of Se employed (260). Generally speaking, the salutary impacts of selenium become particularly noticeable in populations characterized by initially low selenium levels and modest dietary intakes (261, 262). Selenium is considered a hormone chemical, a compound with a biphasic dose response that is toxic at elevated concentrations but has beneficial properties at low doses. Selenium acts as an anti-cancer agent by employing a multifaceted approach to combat malignancy. It impedes the invasive and metastatic capabilities of tumor cells, initiates the process of apoptosis, instigates cell cycle arrest, and fosters DNA repair mechanisms. The anticancer properties of selenium have been well-documented across various types of cancer, including those of the breast, liver, lung, colon, skin, and rectum. Given its broad spectrum of activity and the established evidence of its efficacy, selenium holds considerable promise as a therapeutic agent in the clinical management of cancer.

Currently, a total of 25 selenoproteins have been discovered in humans, most of which act as oxidoreductases with selenocysteine as the catalytic redox activesite. Selenocysteine is a true protein amino acid with a selenol function (SeH) that can be inserted into selenoproteins. Although the biosynthesis of selenocysteine has been characterized as “expensive” and “inefficient”, its incorporation into proteins allows biological systems with the ability to execute fundamental chemical functions that are beyond the specialized capabilities of cysteine (263). Glutathione peroxidases (GPxs), iodothyronine deiodinases, and thioredoxin reductases (TrxRs) all rely on the reactivity of the selenol group within selenocysteine residues to perform their vital functions. These enzymes, including GPxs and TrxRs, play a crucial role in shielding cells from oxidative stress, a primary factor in the initiation and advancement of numerous diseases. Their dependence on the unique chemical properties of the selenol moiety highlights the irreplaceable function of selenium in maintaining cellular health and combating oxidative damage.

Ensuring an adequate intake of selenium may mitigate the risk of developing cancer, autoimmune disorders, infertility issues, or succumbing to severe illnesses, although it is acknowledged that certain conditions arise from specific genotypes of selenoproteins. Under such circumstances, the supplementation with either organic or inorganic forms of selenium is a widely adopted approach to guarantee sufficient selenium levels. Both *in vivo* and *in vitro* research has demonstrated that selenium compounds manifest their anticancer properties through diverse mechanisms. Nevertheless, additional investigative efforts and clinical trials are essential to establish these selenium compounds as recognized anticancer agents in clinical practice. Beyond their application in cancer therapy, selenium compounds have also proven to be valuable in various cancer-related domains, such as chemoprevention, diagnostic procedures, and imaging techniques. Furthermore, their utility extends to areas beyond oncology, highlighting the broad spectrum of potential benefits associated with selenium supplementation.

Recently, there has been a significant upsurge in the interest surrounding the development of nanomaterials with enhanced anticancer activity and less adverse effects on the body as promising candidates for cancer treatment. In light of this burgeoning interest, selenium-containing nanoparticles are under investigation for their potential as novel therapeutic agents in the fight against cancer. We can consider them as potential novel therapeutic agents, both as drug delivery carriers and directly as anticancer protective agents. The potential of selenium nanoparticles in the near future can be considered for pharmaceutical applications and nutritional supplements. There is a pressing need for more rigorous and precise clinical trials to assess the viability and benefits of selenium nanoparticles for enhancing human health. To advance this field, extensive research is imperative to devise synthetic methods that are both less toxic and more cost-effective. Additionally, a deeper understanding of the function of selenium nanoparticles in cancer therapy is necessary, particularly in the context of chemotherapy and radiotherapy. This knowledge will be crucial for optimizing their therapeutic efficiency and managing their cytotoxic effects, thereby harnessing their potential to improve cancer treatment outcomes.

In summary, remarkable advancements have been made over the past decade in understanding the complex biology and chemistry of Se. Se-based cancer therapies may have a bright future, but a great deal of research work is still needed before we see clinical candidates or even approved drugs. This ongoing effort is crucial to transition from theoretical potential to tangible clinical applications, including the development of viable candidates for clinical trials and, ultimately, the approval of new drugs for cancer treatment.
